# The effect of medication on serum anti-müllerian hormone (AMH) levels in women of reproductive age: a meta-analysis

**DOI:** 10.1186/s12902-022-01065-9

**Published:** 2022-06-14

**Authors:** Wei-Wei Yin, Chang-Chang Huang, Yi-Ru Chen, Dan-Qing Yu, Min Jin, Chun Feng

**Affiliations:** Department of Reproductive Medicine, The Second Affiliated Hospital of Zhejiang University School of Medicine, 88 Jiefang Road, Hangzhou, 310009 Zhejiang China

**Keywords:** Anti-müllerian hormone (AMH), Ovarian reserve, Medicine application, Oral contraceptives, Meta-analysis

## Abstract

**Objective:**

The study aims to address whether serum anti-müllerian hormone (AMH) levels fluctuate in the short term after medication application, including oral contraceptives (OCs), metformin (MET), Gonadotropin-releasing hormone agonist (GnRH-a), dehydroepiandrosterone (DHEA), vitamin D (VD), clomiphene citrate (CC), and letrozole (LET).

**Methods:**

Published literature from PubMed, Embase, and Cochrane central was retrieved up until 19 September 2021. A total of 51 self-control studies with an average Newcastle–Ottawa quality assessment scale (NOS) score of 6.90 were analyzed. The extracted data were entered into Stata software, and the weighted mean difference/standardized mean difference (WMD/SMD) and 95% confidence interval (CI) were used for data analysis.

**Results:**

After OCs treatment the AMH level showed a significant decline in women with normal ovarian function, which was significant within 3 months (WMD = -1.43, 95% CI: -2.05 to -0.80, P < 0.00001). After MET treatment, the serum AMH decreased in polycystic ovary syndrome (PCOS) patients (WMD = -1.79, 95% CI: -2.32 to -1.26, *P* < 0.00001), in both obese and non-obese patients. GnRH-a treatment in endometriosis patients led to dynamic changes in the serum AMH levels, that is, ascent at 1 month (*P* = 0.05), and descent at 3 months (*P* = 0.02). After DHEA treatment the serum AMH increased in diminished ovarian reserve (DOR) / poor ovarian response (POR) patients (WMD = 0.18, 95% CI: 0.09 to 0.27, *P* < 0.0001). After VD treatment the serum AMH increased, and it was obvious in non-PCOS patients (WMD = 0.78, 95% CI: 0.34 to 1.21, *P* = 0.0004). After CC treatment the serum AMH decreased significantly in PCOS patients, specifically in non-obese patients (WMD = -1.24, 95% CI: -1.87 to -0.61, *P* = 0.0001).

**Conclusions:**

Serum AMH levels may be affected in the short term after drug application. Specifically, OC, MET and CC lead to decreased AMH level, DHEA and VD lead to increased AMH level, and GnRH-a leads to dynamic variation, which is correlated with PCOS, obesity, age, and duration of medication. The impacts of these medications should be taken into consideration when AMH is used as a marker of ovarian reserve.

**Supplementary Information:**

The online version contains supplementary material available at 10.1186/s12902-022-01065-9.

## Introduction

Anti-Müllerian hormone (AMH) is a dimeric glycoprotein that belongs to the transforming growth factor-β (TGF-β) family [1, 2], and a female baby in the fetal period begins to produce AMH from the 9th month [3]. AMH is secreted by the antral follicles and small antral follicles in the ovary. The greater the number of these follicles, the higher the serum AMH concentration. Because of this feature, AMH is considered to be a marker for the process of ovarian aging [4]. The objectivity and potential standardization of AMH levels, as well as their readily detectable convenience throughout the menstrual cycle, make AMH levels the gold standard biomarker for assessing ovarian reserve and predicting ovarian response to stimulation [5]. It is currently one of the best indicators for assessing ovarian function, guiding assisted reproduction, and indicating iatrogenic damage (such as chemotherapy, radiotherapy or surgery) to the ovarian follicle reserve. It has a broader application in assisted reproduction field [6, 7]. Therefore, the accurate measurement of AMH will guide the dosage of ovarian stimulation-related programs, and it has important reference significance to improve the outcome of assisted reproduction technology [8].

Previous studies believed that AMH was stable and not affected by the menstrual cycle, or hormone drug use. However, more and more clinical studies have shown that drug use may interfere with serum AMH levels in the short term, which may lead to the risk of clinical misinterpretation of AMH values [9]. However, the sample size of relevant research reports is mostly small, the research results often contradict each other, and there is a lack of evidence-based analysis on the subject. Therefore, we carried out a meta-analysis to evaluate the impact of drug use on AMH levels. In the present study, an evidence-based investigation was performed on seven kinds of medications, including oral contraceptives (OCs), metformin (MET), Gonadotropin-releasing hormone agonist (GnRH-a), dehydroepiandrosterone (DHEA), vitamin D (VD), clomiphene citrate (CC), and letrozole (LET), and the variation in serum AMH levels was recorded to guide the correct interpretation and effective application of AMH values in clinics. The findings will provide useful information for elucidating the relationship between the medicine application and the fluctuation of AMH.

## Materials and methods

### Literature search and study selection

This study was based on the PRISMA guidelines [10] for systemic review and meta-analysis. The authors searched PubMed, Cochrane, EMBASE, until September 19th, 2021 and without limitation of region, language, or publication type. Reference list of all selected articles were independently screened to identify additional studies left out in the initial search. Combinations of the following MeSH terms and free words were used: (Anti-Müllerian hormone OR AMH OR MIS OR Müllerian inhibiting substance) AND ((Oral Contraceptives OR Oral Contraceptive OR OC OR COCS) OR (Metformin OR Dimethylbiguanidine OR Glucophage) OR (DHEA OR dehydroepiandrosterone) OR (Gonadotropin Releasing Hormone OR GnRH) OR (Vitamin D OR 25 hydroxyvitamin D) OR (Clomifene OR Chloramiphene OR Clomifen OR Clomiphene Citrate) OR Letrozole). Bibliographies were cross-referenced to identify additional studies. All studies after the search were screened and analyzed by two authors independently (YWW and HCC), and any disagreement will be resolved by discussion until consensus were reached or by consulting the third author (CYR). This paper included prospective self-control studies. Studies were included in this meta-analysis if they met the following criteria: (1) the study population included reproductive-age women; (2) serum AMH were measured in all study participants at least once before and after medication; (3) the association between different drugs and AMH levels was described and quantitative information was provided. Studies were excluded if: (1) Clinical case report, review, meta-analysis or cell, animal model; (2) Evidence-based information comes from books, conferences, notes, thesis, case series, letters, or unpublished studies; (3) unreliable extracted data, overlapped data sets, and paragraphs only abstract available.

### Data extraction

The following data was extracted from every study by two reviewers independently: (1) name of the first author, (2) year of publication, (3) study population and sample size, (4) inclusion and exclusion criteria, (5) age of the subjects (6) AMH assay, (7) study type, (8) mean change of anti-Müllerian hormone. We contacted investigators for additional information when extra information was required.

### Assessment of study quality

We used the Newcastle–Ottawa scale (NOS) to assess the quality of the included literature [11]. The NOS scale was based on 3 indicator systems, including suitable study object selection, inter-group comparability, and intervention exposure. It consisted of 8 indicators, each with a score of 0 or 1, and the “inter-group comparability” can be given 0 or 1 or 2 points, so the overall quality assessment score for each article ranged from 0 to 9 points. Each study was evaluated independently by two authors. Any disagreement was resolved by discussion until consensus reached (Table S8).

### Statistical analysis

All data were entered into Stata (version14.0). Literature heterogeneity was assessed by Q test and quantified by I^2^ index, If values of I^2^ ≤ 25%, it meant that our results were of low heterogeneity. If *P* > 0.10 and 25% < I^2^ < 50%, then the heterogeneity was acceptable. The fixed effects model (FEM) [12] was used to calculate the parameters of the data pool. If *P* < 0.10 and 50% < I^2^ < 75%, then the heterogeneity could not be ignored, and the random effects model (REM) was used to calculate the parameters of the data pool. We performed a subgroup analysis of results with high heterogeneity I^2^ ≥ 75%. Since it was continuous data, the serum AMH variation over time was assessed by calculating the Weighted Mean Difference (WMD) or Standard Mean Difference (SMD) among the pooled data, and the statistical significance was calculated with Z test.

### Publication bias

Publication bias was evaluated by examining the asymmetry of funnel plot. If the scatter points of the documents were symmetrical on both sides of the funnel chart, it indicated that the publication bias of the literature was small, and vice versa, it indicated that the publication bias was large. The literature used in this article was symmetrical on both sides of the funnel chart, indicating that there was no serious publication bias.

### Author contributions

Chun Feng and Wei-Wei Yin designed the study and wrote the paper; Chang-Chang Huang, Yi-Ru Chen and Dan-Qing Yu performed the data curation; Wei-Wei Yin, Chang-Chang Huang, and Min Jin analyzed the data.

## Results

### Study selection and characteristics of included studies

**Fig. 1:**
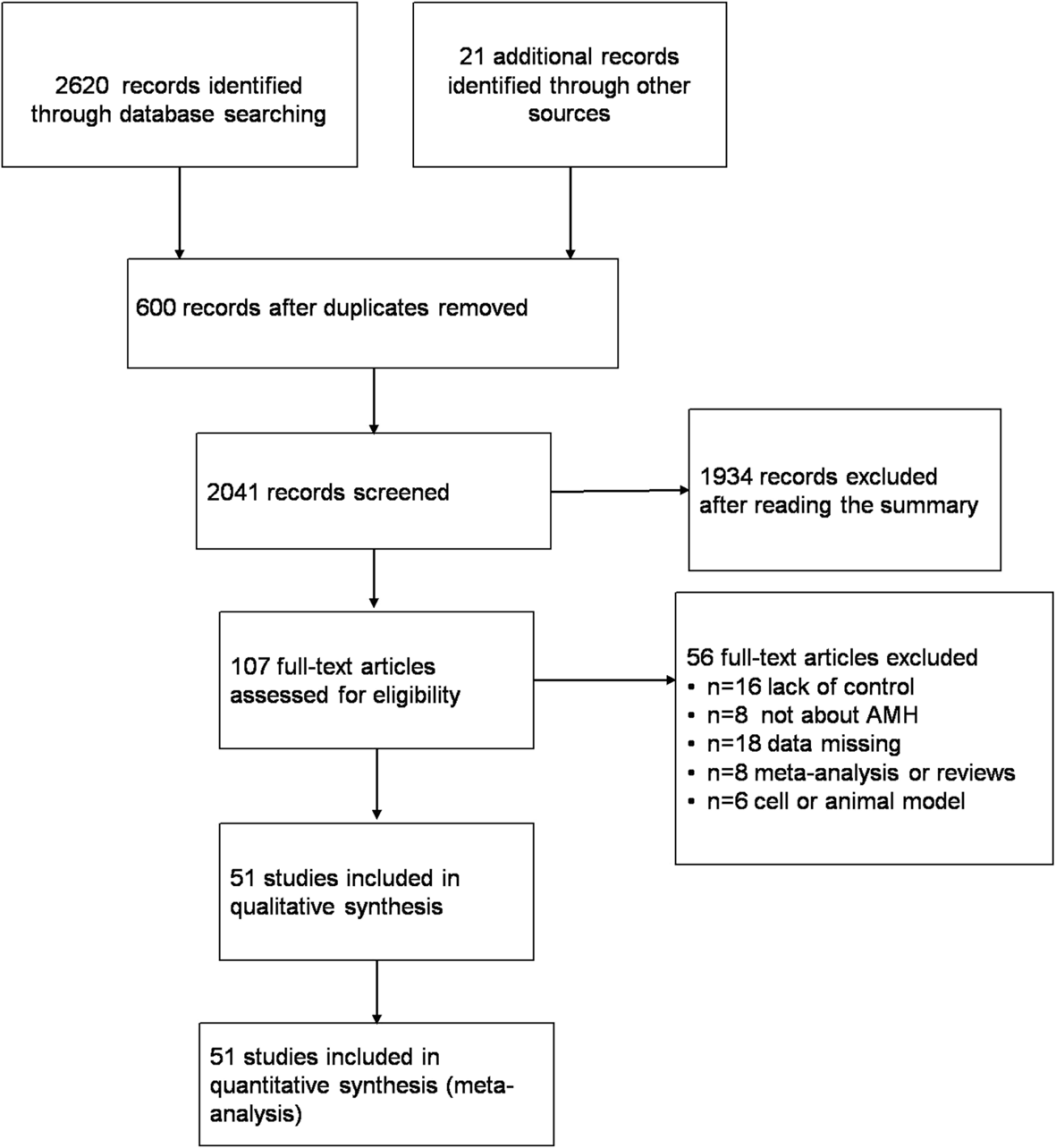
Flow chart for the selection of the retrieved articles

A total of 2620 articles were recognized by database searching and 21 through other sources. 600 duplicated records were removed, and 1934 studies were excluded based on information from titles and abstracts. Due to the exclusion reasons listed in the flow chart, 51 studies remained for the qualitative synthesis. Figure 1 shows the study flow diagram of the searching process of these records.

### Meta-analysis results

META analysis showed the trend of serum AMH changes after the application of 7 drugs as shown in the table below (Table 1).

**Table 1:**
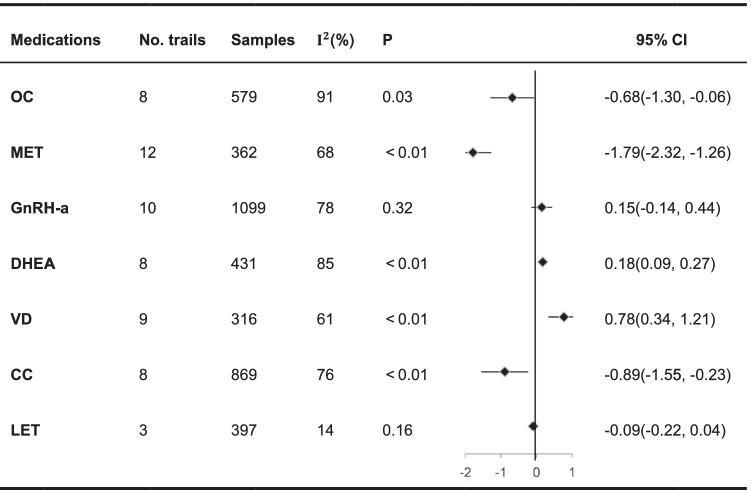
Overall meta-analysis of the effects of 7 drugs

Negative values in forest plot: AMH value decreased after medication; positive values in the forest plot: AMH value increased after medication

#### Variation of serum AMH levels in women with normal ovarian function after taking OCs

Women with normal ovarian function taking OC (conventional artificial cycle medication: 1 capsule per day for 21 days, repeated 7 days after stopping the drug; or continuous medication: 1 capsule per day, uninterrupted) 3 ~ 6 cycles or more than 6 cycles, a total of 9 articles [13–20] were included (the total number of sample cases *n* = 579, 8 groups self-control studies) used for the analysis of this topic. (Table S1).

REM analysis of all 8 sets of data (*n* = 579) showed that women with normal ovarian function taking OC (3–6 cycles or more than 6 cycles) have a significant downward trend in serum AMH (WMD: -0.68,95%CI: -1.30 to -0.06; *P* = 0.03). The decrease in serum AMH level was statistically significant.

**Fig. 2:**
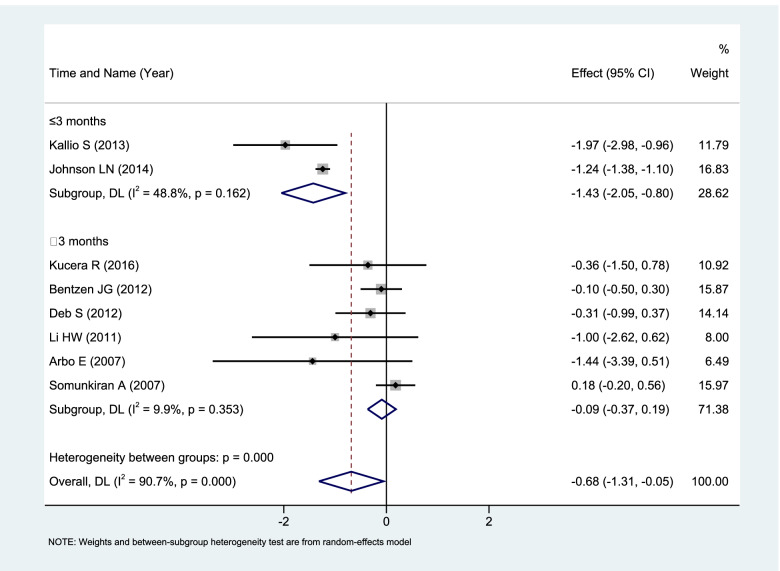
Forest plot of Meta subgroup analysis of serum AMH level changes in women with normal ovarian function taking OCs (≤ 3 months vs. > 3 months)

Subgroup analysis was performed according to the duration of OCs. Serum AMH level decreased significantly (WMD: -1.43, 95%CI: -2.05 to -0.80; *P* < 0.00001) in the subgroup of use time ≤ 3 months (*n* = 165). With use for more than 3 months or even longer, there was no significant effect on serum AMH levels (WMD: -0.09, 95%CI: -0.37 to 0.19; *P* = 0.45) see Fig. 2.

#### Variation of serum AMH levels in PCOS patients with MET pretreatment

Regarding PCOS patients with MET pretreatment (conventional medication 1500 ~ 2250 mg, 2 ~ 3 times a day orally, continuous medication for 2 ~ 12 months), a total of 12 articles [21–32] were included (total number of sample cases *n* = 362, 12 groups of self-control studies) (Table S2).

REM analysis of all 12 sets of data (*n* = 362) showed that MET (2–12 months) led to a significant decrease in serum AMH in PCOS patients. (WMD: -1.79, 95%CI: -2.32 to -1.26,*P* < 0.00001).

**Fig. 3:**
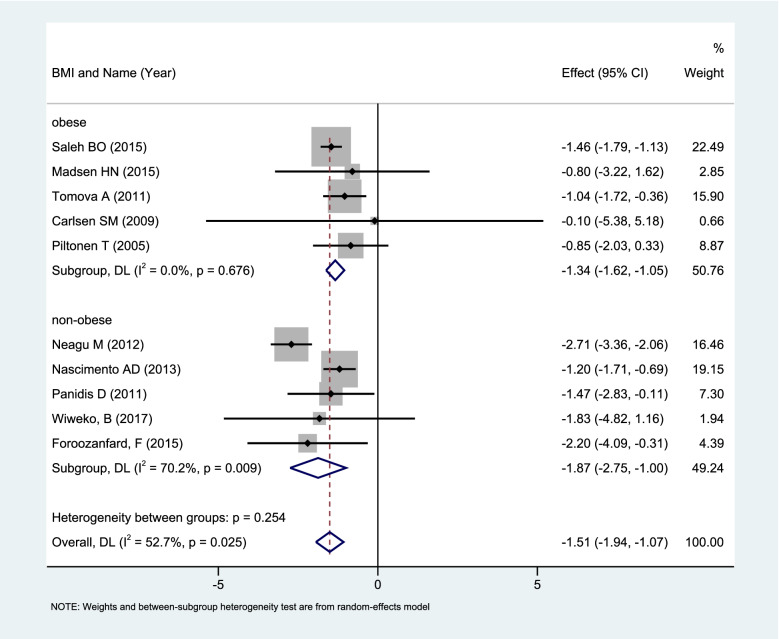
Forest plot of Meta subgroup analysis of changes in serum AMH levels of non-obese vs obese PCOS patients with MET pretreatment

The above mentioned 12 groups of research data were highly heterogeneous (I2 = 68%, *P* = 0.0003). The meta subgroup analysis of a random effect model based on whether they were obese (BMI > 30 kg/m2) showed that obese patients MET pretreatment (*n* = 151, 5 sets of data) caused a significant decrease in serum AMH levels (WMD: -1.34, 95%CI: -1.62 to -1.05, *P* < 0.00001). Corresponding non-obese patients MET pretreatment (*n* = 126, 5 sets of data) could also cause a significant decrease in serum AMH levels (WMD: -1.87, 95%CI: -2.75 to -1.00; *P* < 0.0001) (Fig. 3).

#### Variation of serum AMH levels in endometriosis patients GnRH-a pretreatment

Regarding endometriosis patients GnRH-a pretreatment (conventional medication: One injection of long-acting (3.75 mg per tube) leuprolide for 7–21 days during menstruation, or short-acting leuprolide (0.1 mg per tube) daily from 7–21 days of menstruation to the day of ovulation induction), a total of 5 articles [33–37] were included, with 10 sets of data (the total number of sample cases *n* = 1099, 10 groups of self-control studies) (Table S3).

**Fig. 4:**
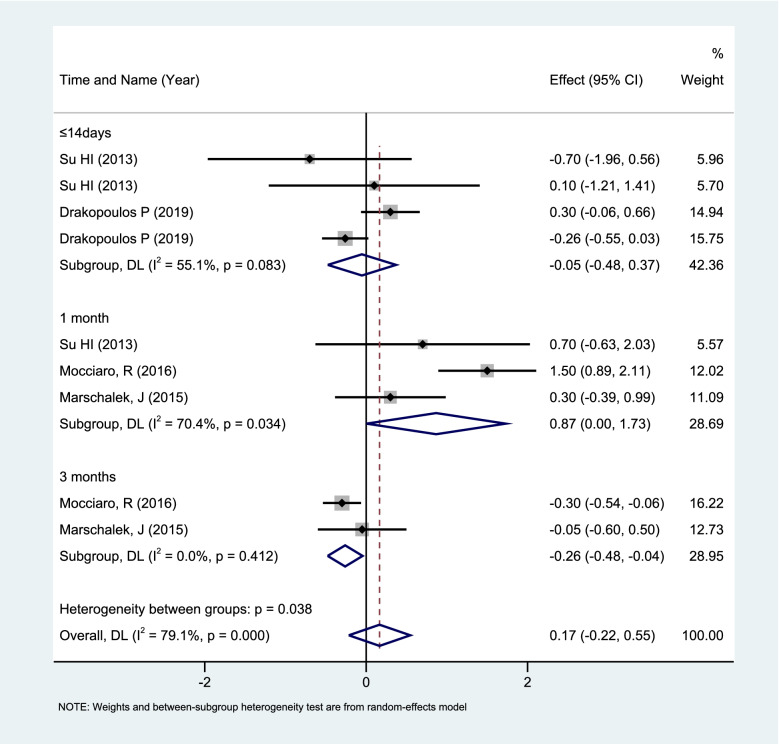
Forest plot of Meta subgroup analysis of changes in serum AMH levels of ≤ 14 days vs. 1 month vs. 3 months with GnRH-a pretreatment

REM analysis of all 10 sets of data (*n* = 1099) showed that GnRH-a pretreatment (7 days to 6 cycles) can cause dynamic changes in serum AMH levels in endometriosis patients. Subgroup analysis was performed in 9 groups according to the blood collection time of the subjects after GnRH-a pretreatment (≤ 14 days, 1 month, 3 months), which showed that the use of GnRH-a for a short period of time(≤ 14 days) had little effect on the serum AMH levels. After 1 month, there was a transient increase (WMD: 0.87; 95%CI: 0.00 to 1.73; *P* = 0.05), and the serum AMH decreased after 3 months (WMD: -0.26; 95%CI: -0.48 to -0.04; *P* = 0.02) See Fig. 4.

#### Variation of serum AMH levels in DOR/POR patients DHEA (dehydroepiandrosterone) pretreatment

**Fig. 5:**
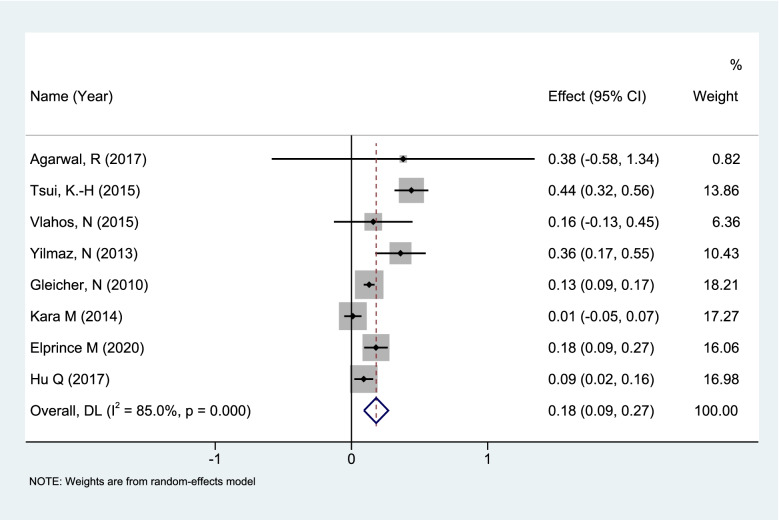
Changes in serum AMH levels in DOR/POR patients taking DHEA (dehydroepiandrosterone)

For the effect of DHEA pretreatment on AMH in DOR/POR patients (conventional medication 75 mg, 3 times a day) a total of 8 articles [38–45] (total number of sample cases *n* = 431, 8 groups of self-control studies) were included (Table S4). REM analysis of 8 sets of data (*n* = 431) showed that DHEA led to a significant increase in serum AMH (WMD: 0.18, 95% CI:0.09 to 0.27; *P* < 0.0001) (Fig. 5).

#### Variation of serum AMH levels in women with VD pretreatment

**Fig. 6:**
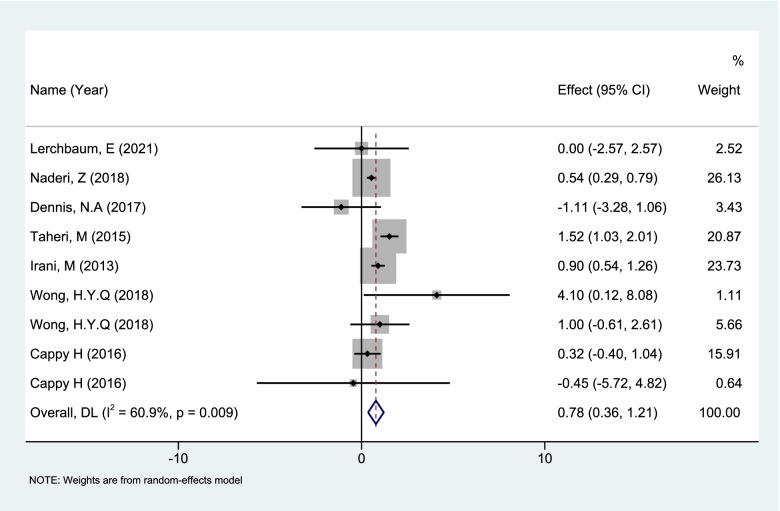
Forest plot of Meta analysis of changes in serum AMH levels after VD pretreatment

Regarding VD pretreatment (conventional medication 2000 IU-5000 IU/week, continuous medication for 2 weeks to 6 months), a total of 7 articles [46–52] were included (total number of sample cases *n* = 316, 9 groups of self-controlled studies) (Table S5). REM analysis of 9 sets of data (*n* = 316) showed that VD pretreatment (2 weeks to 6 months) in patients caused an increase in serum AMH (WMD: 0.78, 95%CI: 0.34 to 1.21; *P* = 0.0004) (Fig. 6).

A subgroup analysis was performed according to whether they were PCOS patients. In PCOS patients VD supplementation could cause the fluctuate of serum AMH levels (WMD: 1.16, 95% CI: -1.58 to 3.89; *P* = 0.41), but this fluctuation was not statistically significant. In non-PCOS patients VD supplementation caused a statistically significant increase in serum AMH (WMD: 0.77, 95%CI: 0.33 to 1.21; *P* = 0.0007).

#### Variation of serum AMH levels PCOS patients with CC pretreatment

Regarding CC pretreatment in PCOS patients (conventional medication 50 mg/day, continuous medication for 1 to 3 cycles), a total of 8 articles [53–60] were included (total number of sample cases *n* = 869, 8 groups of self-control studies) for the analysis of this topic (Table S6). REM analysis of 8 sets of data (*n* = 869) showed that CC pretreatment (1 ~ 3 cycles) can cause a significant decrease in serum AMH levels in PCOS patients (WMD: -0.89, 95%CI: -1.55 to -0.23; *P* = 0.008).

**Fig. 7:**
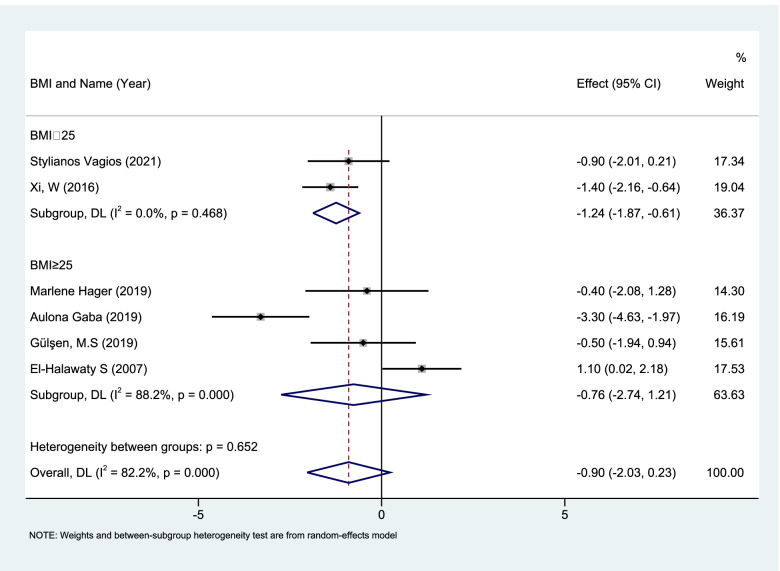
Forest plot of Meta subgroup analysis of changes in serum AMH levels of non-obese vs obese PCOS patients with CC pretreatment

According to whether the study subjects were obese, a REM subgroup analysis showed that in non-obese (BMI < 25 kg/m^2^) patients CC pretreatment (*n* = 376, 2 sets of data) caused a significant reduction in serum AMH levels (WMD: -1.24, 95%CI: -1.87 to -0.61; *P* = 0.0001). There was no significant difference in obese (BMI ≥ 25 kg/m^2^) patients (*n* = 261, 4 sets of data) (Fig. 7.)

#### Variation of serum AMH levels with LET pretreatment

Regarding LET pretreatment (conventional artificial cycle medication 2.5 mg/day, starts on menstrual cycle 3–5 days, continuous use for 5 days for 3 to 6 cycles), a total of 3 articles [55, 56, 61] (total number of sample cases *n* = 397, 3 groups of self-controlled studies) were included (Table S7). FEM analysis of 3 sets of data (*n* = 397) showed that LET (3–6 cycles) have no significant effect on AMH levels in the short term (WMD: -0.09, 95%CI: -0.22 to 0.04; *P* = 0.16).

### Sensitivity analysis

Multi-group meta-analysis of literature sample data in this study showed significant heterogeneity (I^2^ > 50%), so the sensitivity analysis was performed by removing one study at one time. For the above 7 different drug sample data, we found that removing any study in the analysis did not impact the overall results, which indicated that the meta-analysis results of the corresponding group were stable.

## Discussion

In assisted reproduction clinics, OCs is often used as a pretreatment medication before ovarian stimulation. OCs can negatively inhibit the secretion of FSH and LH, adjust the menstrual cycle, improve women’s ovarian response and assisted reproduction outcomes. Traditional studies believed that after OCs application there have no significant effect on serum AMH levels in the short term [17, 20, 62], but some recent research results did not support the conclusion [14, 15]. The influence of AMH level was related to the dosage, type of contraceptives and time of administration, female age, self-condition and so on.

The results of this study support that OCs pretreatment in women with normal ovarian function has a downregulation effect on serum AMH. The duration of drug use, the age of the subjects, the degree of obesity may be the source of heterogeneity in this study, and the subgroup analysis according to the medication use duration shows that heterogeneity decreased. The downregulation effect of OCs is obvious in the short term of medication. AMH is secreted by prefollicles and antral follicular granulosa cells which are sensitive to FSH. The down-regulation of FSH caused by OCs reduce the stimulation of granulosa cells, which will have a down-regulating effect on the secretion of AMH [17, 63]. However, with the extension of use time, the decrease of serum AMH decreases. This may due to the granule cells adaptation to the down-regulation of FSH to a certain degree, or the concentration of AMH may differ greatly from different experiments, so statistical uncertainty increase. In clinical practice, some PCOS patients who used OCs can ovulate spontaneously within a short time after stopping the drug, which may be related to the down-regulation of AMH by OC, reduced the inhibition of follicular development. This meta-analysis further confirmed that the serum AMH concentration in women who use hormonal contraception would be negatively affected by exogenous sex hormones, and may not be able to maintain its value as a predictor of ovarian reserve, therefore, we recommend women who use OCs to measure their serum AMH levels at least 3 months after stopping the drug.

PCOS is one of the most common causes of female infertility, affecting about 8% of women in childbearing age. The increase in serum AMH in women with hyperandrogenism and/or oligoovulation may indicate the presence of PCOS. Serum AMH is a useful prognostic biochemical marker for MET treatment in PCOS. As a first-line treatment for insulin resistance, MET can improve insulin sensitivity and regulate blood sugar levels, thereby alleviating insulin resistance, which can also reduce androgen levels and improve ovulation [64]. Currently, MET has become a commonly used drug before assisted reproduction in women with PCOS.

In this article, a meta-analysis of 12 groups of PCOS patients taking MET suggested that: the use of MET in PCOS patients will cause a decrease in serum AMH levels, both obese (BMI ≥ 30 kg/m^2^) and non-obese (BMI < 30 kg/m^2^) patients can occur, which suggests that even patients who are not obese can use MET to reduce the level of AMH, reduce the inhibition of follicular development, and increase the chance of spontaneous ovulation.

Endometriosis is a chronic estrogen-dependent disease. Common symptoms include secondary dysmenorrhea, dyspareunia, chronic pelvic pain and infertility. Although the exact mechanism leading to infertility is still unclear, some studies suggest that the excessive production of inflammatory cytokines, growth factors, and chemokines in endometriosis may cause the inflammation process to damage the ovaries, fallopian tubes and endometrial functions [65, 66]. GnRH-a are common treatments for endometriosis. They inhibit the production of hypothalamic-ovarian axis and ovarian steroids, leading to a decrease in estrogen levels. In addition, they also reduce the expression of growth factors which participate in endometriosis tissue development, such as vascular endothelial growth factor (VEGF), and minimize the macrophage infiltration and micro vessel density of endometriosis lesions [37, 67]. Studies have shown that in women with infertility related to endometriosis, given GnRH-a 3–6 months before in vitro fertilization (IVF) or intracytoplasmic sperm injection (ICSI) can significantly improve assisted reproduction outcomes [68], but the effect of GnRH-a on serum AMH levels is still controversial.

In this study, the dynamic observation of serum AMH after GnRH-a use emphasized the complexity of AMH levels after GnRH-a use. The heterogeneity in the study results may be related to the duration of GnRH-a use, the age of the subjects, and the severity of the endometriosis. Heterogeneity between groups was reduced after subgroup analysis according to duration of GnRH-a use. Serum AMH levels did not change much within 14 days, but some studies pointed out that there was a brief drop in serum AMH levels due to the up-regulation of GnRH receptors, and the anti-proliferation and apoptosis effects of GnRH-a short-term exposure on granulosa cells [69]. At the same time, the short term decrease in AMH may lead to the enlargement of the follicular pools of the anterior and small sinuses that secrete AMH, causing an increase in AMH levels on the 1 month [33, 70]. Our study emphasized that after using GnRH-a, AMH levels follow a predictable two-way trajectory, which also limits the application of AMH as a marker of ovarian reserve in the past 3 months after treatment, so it is recommended to perform AMH test after stopping the drug for more than 3 months to determine the ovarian reserve function.

DOR/POR is a recognized state of ovarian failure [71], and is one of the most challenging problems in artificial reproductive medicine. DHEA is not only a food supplement, naturally found in wild yam and soy products, but also a steroid with both androgenic and weak estrogenic activity, which can improve ovarian response, reduce miscarriage and aneuploidy, and increase the chance of live birth [43, 72–74]. The reason is that oocytes are in a resting phase in unrecruited primordial follicles, and once recruited they enter an age-dependent ovarian environment where the follicles mature. The quality of this environment deteriorates evenly as women aged, and affects the separation process of meiosis, leading to aneuploidy. DHEA may change and restore the ovarian environment to prevent the aging of follicles [75]. Other studies have shown that DHEA can increase insulin-like growth factor-1 (IGF-1), promote follicle formation, enhance the effect of gonadotropin and reduce follicular atresia [71, 76–78], and make the outcome of assisted pregnancy significantly improve.

In this article, the meta-analysis of 8 groups of DOR/POR patients taking DHEA suggested that the use of DHEA in DOR/POR patients may cause an increase in serum AMH levels, and this rising effect is obvious in the short term. With the high incidence and severity of DOR/POR in aged patients, whether DHEA pretreatment can achieve the same effect for this type of patients was another aspect. Previous studies have shown that patients less than 35 years old after pretreatment with DHEA, whether the number of follicles obtained, the fertilized eggs, or the serum E2, FSH, LH, or AMH level, all better than women more than 35 years old [79, 80]. In this study, in our subgroup analysis of DOR/POR patients by age, we found heterogeneity greatly reduced( I^2^ = 0) among women of advanced reproductive age (> 38 years old), and DHEA pretreatment can cause a significant increase in serum AMH levels (*P* < 0.00001), which suggested that it is also necessary to supplement DHEA for such patients.

VD is a steroid hormone that has a well-known effect on calcium and bone metabolism. The current research has more and more evidence that the concentration of 25-hydroxyvitamin D (25(OH)D) is related to various conditions, including obesity, metabolic disorders [81, 82], cardiovascular disease [83], gonadal function decrease [84], PCOS [85] and decreased female fertility [86]. Studies have shown that VD deficiency was associated with various manifestations of PCOS, including anovulation, hyperandrogen and insulin resistance [87]. VD supplementation has been shown to improve menstrual cycles, hyperandrogen and metabolic disease in PCOS [88, 89], which shows that VD has a direct impact on female fertility.

The 9 sets of data in this article show that in non-PCOS patients serum AMH level increases in the short term after VD pretreatment, but in PCOS patients this increase is not obvious. The results of this meta-analysis demonstrate that the relationship between VD and AMH is complex. Heterogeneity in the populations studied may account for some of the conflicting data reported, since some studies were carried out in normal non-infertile ovulatory women while others were in women with PCOS. In addition, VD levels are also affected by such as race, region and season (sun exposure). These may be important sources of heterogeneity in the findings. According to our subgroup analysis results, we encourage non-PCOS patients to supplement VD appropriately. Meanwhile, there is no need to worry about the increase of AMH after VD administration for the patients with PCOS.

Similarly, as a common endocrine disease, PCOS affects 6–10% of women of childbearing age [90]. Sparse ovulation or anovulation caused by PCOS is a common cause of infertility. CC as a first-line drug for inducing ovulation is widely used in ovulation therapy [90, 91]. It is a selective estrogen receptor modulator that can antagonize the negative feedback of endogenous estrogen on the hypothalamic-pituitary axis. CC treatment can restore luteinizing hormone to normal, increase the secretion of follicle stimulating hormone, thereby promoting follicular growth and ovulation [92], and increase the chance of ovulation and conception in PCOS patients. In addition, existing studies have shown that obesity is an important parameter, which will have a negative impact on the response of PCOS patients to CC [93].

The research results of CC pretreatment in 8 groups of PCOS patients in this article all indicate that the AMH levels have a short-term reduction after using CC, and it is more obvious in non-obese patients, so we can assume that thinner people represent better sensitivity to CC responses.

As an ovulation-stimulating drug, LET was initially used in Clomiphene -resistant cases. In recent years, evidence has shown that compared with CC, LET stimulation has a higher ovulation rate, pregnancy rate, cumulative live birth rate, and lower multiple births [94–96]. The present analysis shows that serum AMH levels are not significantly affected after LET use.

The present study is the first meta-analysis addressing the effect of multiple medications on AMH levels, including OCs, MET, GnRH-a, DHEA, VD, CC, and LET. By studying the effects of seven drugs on serum AMH values, our study can provide effective guidance for explanation of AMH values in clinical practice, and is meaningful for the prediction of ovarian function in different groups of women.

Limitations are as follows. Firstly, since we failed to connect with some authors to collect some original data, the power of the subgroup analysis of GnRH-a might be compromised. Secondly, although we retrieved relevant articles from multiple databases, there are still some unpublished data that we don’t have access to. Thirdly, original studies used various control groups, including healthy women, infertile women, elderly women and various diagnostic criteria of PCOS, which makes it difficult to control the confounding factors.

## Conclusion

Medication application may affect serum AMH levels in the short term. Specifically, OC, MET and CC lead to decreased AMH level, DHEA and VD lead to increased AMH level, and GnRH-a leads to dynamic variation, which is correlated with PCOS, obesity, age, and duration of medication. The impacts of these medication should be taken into consideration when AMH is used as a marker of ovarian reserve.

## Supplementary Information

**Additional file 1:** **Table S1. **The characteristics of the studies included for qualitative analyses.

**Additional file 2:** **Table S2.** The characteristics of the studies included for qualitative analyses.

**Additional file 3:** **Table S3.** The characteristics of the studies included for qualitative analyses.

**Additional file 4:** **Table S4. **The characteristics of the studies included for qualitative analyses.

**Additional file 5:** **Table S5. **The characteristics of the studies included for qualitative analyses.

**Additional file 6:** **Table S6. **The characteristics of the studies included for qualitative analyses.

**Additional file 7:** **Table S7.** The characteristics of the studies included for qualitative analyses.

**Additional file 8:** **Table S8**. Newcastle-Ottawa Scale for assessing quality of interventional cohort studies.

## Data Availability

All data analyzed during this study are included in the supplementary information tables (Tables S1, S2, S3, S4, S5, S6 and S7).
