# Supplementary material for: The effect of medication on serum anti-müllerian hormone (AMH) levels in women of reproductive age: a meta-analysis
Source: BMC Endocr Disord. 2022 Jun 14;22:158. doi: 10.1186/s12902-022-01065-9 (PMC9195431; doi:10.1186/s12902-022-01065-9)
Supplement: Supplementary file 1 — Additional file 1: Table S1. The characteristics of the studies included for qualitative analyses. [file 12902_2022_1065_MOESM1_ESM.docx]

**TABLE S1** The characteristics of the studies included for qualitative analyses.

| **Study** | **Year** | **Inclusion criteria** |  |  |  | **Study type** | **OC** | **Serum AMH level**  **(ng/ml)** | |
| --- | --- | --- | --- | --- | --- | --- | --- | --- | --- |
|  |  |  | **Population** | **Age**  **(range, mean or media)** | **AMH Assay** |  |  | **Before** | **After** |
| Kucera R ^[13]^ | 2016 | Women use OC at least 10 years | 105 healthy women | 32.1 | ECL | Self-control | Long-term | 3.91±3.37 | 3.55±2.89 |
| Johnson LN ^[14]^ | 2014 | Women use OC within the preceding 3months | 123 healthy women | 25.5 | Gen II ELISA | Self-control | 3 m | 2.32±0.45 | 1.08±0.66* |
| Kallio S ^[15]^ | 2013 | Women treat with combined OCs | 42 healthy women | 20-33 | Gen II ELISA | Self-control | 2 m | 3.88±3.0 | 1.91±1.5* |
| Bentzen JG ^[16]^ | 2012 | Use combined OCs | 217 healthy  female healthcare workers | 29.6-32.6 | Immunotech  ELISA | Self-control | Long-term | 2.9±2.2 | 2.8±2.08 |
| Deb S ^[17]^ | 2012 | non-smoking status, regular menstrual cycle and no past history of ovarian surgery | 34 voluntee-rs recruited | 18-35 | MIS/AMH ELISA kit | Self-control | 1 y | 3.06±1.27 | 2.75±1.59 |
| Li HW ^[18]^ | 2011 | non-pregnant state, none using any hormonal medication within  3 months | 23 healthy women recruited  at the Birth Control Clinic | 30  (26-35) | Immunotech  ELISA | Self-control | 4 m | 3.5±3.3 | 2.5±2.2* |
| Arbo E ^[19]^ | 2007 | regular 28 to 34 day menses; ≤35 age; both ovaries present; absence of any endocrinological or ovulation disorder;  BMI18-25kg/m2; absence of any hormonal therapy in the past 3 months | 20 normoovul-atory infertile women | 29.1±4.11 | DSL ELISA | Self-control | Long-term | 3.5±4.1 | 2.06±1.7* |
| Somunkiran A ^[20]^ | 2007 | Regular menstrual cycle no evidence of androgen excess | 15 women with normal menstrual cycles | 24.8±5.71 | MIS/AMH ELISA kit | Self-control | 6 m | 1.93±0.51 | 2.11±0.56 |

OC: oral contraceptive; BMI, body mass index; ELISA, enzyme-linked immunosorbent assay; ECL: Electro Chemi-Luminescence; DSL: Diagnostic Systems Laboratories; OC (Regular): 1 capsule per day for 21 days, repeated 7 days after stopping the medication; OC (Long-term): 1 capsule per day, uninterrupted; M: months; Y: year; *: Before vs. After P < 0.05; Serum AMH level: Mean ± SD or media (95%CI).
