# Supplementary material for: The effect of medication on serum anti-müllerian hormone (AMH) levels in women of reproductive age: a meta-analysis
Source: BMC Endocr Disord. 2022 Jun 14;22:158. doi: 10.1186/s12902-022-01065-9 (PMC9195431; doi:10.1186/s12902-022-01065-9)
Supplement: Supplementary file 2 — Additional file 2: Table S2. The characteristics of the studies included for qualitative analyses. [file 12902_2022_1065_MOESM2_ESM.docx]

**TABLE S2** The characteristics of the studies included for qualitative analyses

| **Study** | **Year** | **Exclusion criteria** |  |  |  | **Study type** | **BMI** | **Serum AMH level**  **(ng/ml)** | |
| --- | --- | --- | --- | --- | --- | --- | --- | --- | --- |
|  |  |  | **Population** | **Age**  **(range, mean or media)** | **AMH Assay** |  |  | **Before** | **After** |
| Saleh BO^[21]^ | 2015 | pregnant or diabetic | PCOS women (n=20) | 27.5±1.32 | ELISA | Self-control | 30.85±0.95 | 4.49±0.54 | 3.03±0.53* |
| Madsen HN^[22]^ | 2015 | peri-climacteric gonadotropin values, hyperprolactinemia,  diabetes mellitus, impaired thyroid, renal or hepatic function, hormonal treatment, pregnancy | 33 patients with PCOS | 18-45 | Beckman-Coulter Gen II ELISA | Self-control | ≥30 | 7.4±6.2 | 6.6±5.5 |
| Neagu M^[23]^ | 2012 | None | 11 patients with infertility and PCOS | 20-33 | NA | Self-control | ≥25 | 8.99±0.99 | 6.28±0.46* |
| Nascimento AD^[24]^ | 2013 | PCOS without IR or non-PCOS | PCOS and IR patients (n=16) selected at hospital | 26.3±1 | Beckman-Coulter ELISA | Self-control | 29.1±1.7 | 7.0±0.85 | 5.8±0.78* |
| Tomova A^[25]^ | 2011 | Amenorrhea ＜3 months, no signs of hyperandrogenism,  and polycystic ovaries on ultrasound examination | 22 women with PCOS | 26.5±1.1 | Immunotech ELISA | Self-control | 30.73±1.9 | 6.40±1.30 | 5.36±0.96 |
| Panidis D^[26]^ | 2011 | BMI＞25 kg/m2；Other common causes of hyperandrogenism (prolactinoma,  congenital adrenal hyperplasia, Cushing syndrome and  virilizing ovarian or adrenal tumours) | 15 women with PCOS present at outpatient  endocrine clinic | 20.53±3.09 | ELISA (Nichols Institute Diagnostics, CA) | Self-control | 21.97±1.69 | 9.24±3.70 | 7.77±2.82 |
| Carlsen SM^[27]^ | 2009 | pregnancy, breastfeeding, known liver disease  or alanine  aminotransferase ＞60IU/l；creatinine ＞130 mmol/l, known  alcohol abuse, diabetes mellitus and treatment with oral glucocorticoids  or hormonal contraceptives. | 20 PCOS women from University Hospital | 30.6±5.9 | ELISA | Self-control | 33.4±7.5 | 15.3±11.5 | 15.2±12.0 |
| Piltonen T^[28]^ | 2005 | Suffering from other medical conditions or treated with medication  (including oral contraceptive) | 26 women with PCOS | 26-41 | Beckman-Coulter ELISA | Self-control | 30.1±0.5 | 12.25±2.1 | 11.4±2.24* |
| Chhabra N^[29]^ | 2018 | Women with other causes of PCOS; AMH <5 ng/ml; contraindications to use of insulin; treated with OC or any other insulin  Sensitizer | 35 Infertile patients with PCOS | 30.03±3 | Peripheral venipuncture | Self-control | NA | 11.87±5.6 | 7.4±3.9* |
| Dawoud Z^[30]^ | 2018 | Not stated | 50 PCOS women | NA | NA | Self-control | NA | 15.65±4.78 | 11.43±4.03* |
| Wiweko, B^[31]^ | 2017 | Pregnant women, lactating mothers, patients who consumed ISAs or oral contraceptive pills, Cushing syndrome, late onset congenital adrenal hyperplasia, androgen-secreting tumor, uncontrolled thyroid disease, hyperprolactinemia, or other chronic diseases | 20 PCOS women of reproductive age | 18-40 | Beckman-Coulter Gen II ELISA | Self-control | 28.02±6.02 | 9.30±5.06 | 7.47±4.59* |
| Foroozanfard, F^[32]^ | 2015 | Not stated | 30 infertile women with PCOS | 25.2±4.2 | ELISA | Self-control | 26.2±3.8 | 10±3.75 | 7.8±3.7* |

IR: insulin resistance; OC: oral contraceptive; BMI, body mass index, The unit of BMI: Kg/m2; ELISA: enzyme-linked immunosorbent assay; MET: Metformin; MET (Regular): 1500mg or 2250mg take orally 2-3 times a day; *: Before vs. After P < 0.05; Serum AMH level: Mean ± SD or media (95%CI); BMI: Mean ± SD or media (95%CI); NA: not available.
