# Supplementary material for: The effect of medication on serum anti-müllerian hormone (AMH) levels in women of reproductive age: a meta-analysis
Source: BMC Endocr Disord. 2022 Jun 14;22:158. doi: 10.1186/s12902-022-01065-9 (PMC9195431; doi:10.1186/s12902-022-01065-9)
Supplement: Supplementary file 3 — Additional file 3: Table S3. The characteristics of the studies included for qualitative analyses. [file 12902_2022_1065_MOESM3_ESM.docx]

**TABLE S3** The characteristics of the studies included for qualitative analyses

| **Study** | **Year** | **Exclusion criteria** | **Population** | **Age**  **(range, mean or media)** | **AMH assay** | **Measure time** | **Leuprolide** | **Serum AMH level (ng/ml)** | |
| --- | --- | --- | --- | --- | --- | --- | --- | --- | --- |
|  |  |  |  |  |  |  |  | **Before** | **After** |
| Su HI ^[33]^ | 2013 | pregnancy, lactation, hot flashes, hormonal contraception within 3 months, abnormal  prolactin or thyroid hormone levels | endometriosis women with monthly menstrual cycles (n=33) | 30.3±8.5 | Gen II ELISA | After 7 d | long-acting | 2.4±2.9 | 1.7±2.3* |
| Su HI ^[33]^ | 2013 |  |  |  |  | After 14 d | long-acting | 2.4±2.9 | 2.5±2.5* |
| Su HI ^[33]^ | 2013 |  |  |  |  | After 1 m | long-acting | 2.4±2.9 | 3.1±2.6* |
| Drakopoulos P ^[34]^ | 2019 | AFC ＜7 ; baseline  serum AMH＜1.1 ng/mL. | endometriosis women with normal ovarian responders (n=52) | 36(33-38) | ELISA | After 7 d | short-acting | 1.58±0.84 | 1.32±0.64* |
| Drakopoulos P ^[34]^ | 2019 |  |  |  |  | After 14 d | short-acting | 1.58±0.84 | 1.88±1.04* |
| Cai J ^[35]^ | 2018 | history of surgical treatment on ovary;  abnormal findings on  thyroid function test or elevated prolactin level, and current  medication for chronic diseases | Non-PCOS women (n=714) | 30(28-33) | ELISA | After 14 d | long-acting | 2.9±1.5 | 3.04±1.58* |
| Mocciaro R ^[36]^ | 2016 | None | endometriosis women(n=69) | 31.9±5.6 | NA | After 1 m | long-acting | 2.3±0.6 | 3.8±2.5* |
| Mocciaro R ^[36]^ | 2016 |  |  |  |  | After 3 m | long-acting | 2.3±0.6 | 2±0.8* |
| Marschalek J ^[37]^ | 2015 | hyperandrogenemia was present, untreated hyper- or hypothyroidism,previous GnRH-a application. | women with infertility and endometriosis  (n=22) | 30  (27-35.3) | ELISA DSL | After 1 m | long-acting | 1.2±1.0 | 1.5±1.3 |
| Marschalek J ^[37]^ | 2015 |  |  |  |  | After 3 m | long-acting | 1.2±1.0 | 1.15±0.85 |

PCOS: Polycystic Ovary Syndrome; ELISA, enzyme-linked immunosorbent assay; the usage of leuprolide: Long-acting: leuprolide injection once every four weeks (3.75mg); short-acting: leuprolide (0.1mg) daily from 7-21 days of menstruation to the day of ovulation induction; M: months; D: days; *: Before vs. After P < 0.05; Serum AMH level: Mean ± SD or media (95%CI); NA: not available.
