# Supplementary material for: The effect of medication on serum anti-müllerian hormone (AMH) levels in women of reproductive age: a meta-analysis
Source: BMC Endocr Disord. 2022 Jun 14;22:158. doi: 10.1186/s12902-022-01065-9 (PMC9195431; doi:10.1186/s12902-022-01065-9)
Supplement: Supplementary file 4 — Additional file 4: Table S4. The characteristics of the studies included for qualitative analyses. [file 12902_2022_1065_MOESM4_ESM.docx]

**TABLE S4** The characteristics of the studies included for qualitative analyses.

| **Study** | **Year** | **Exclusion criteria** | **Population** | **Age**  **(range, mean or media)** | **AMH**  **Assay** | **Serum AMH level**  **(ng/ml)** | |
| --- | --- | --- | --- | --- | --- | --- | --- |
|  |  |  |  |  |  | **Before** | **After** |
| Agarwal R ^[38]^ | 2017 | PCOS, premature ovarian failure, hormone-dependent disorders, liver disease, diabetes or  psychiatric illness | 20 patients with high-risk factors for  DOR | 33.10±4.29 | Gen II ELISA | 1.15±1.49 | 1.53±1.62* |
| Tsui K.-H ^[39]^ | 2015 | patients with a history of the following clinical situations: ovarian  cystectomy, oophorectomy, exposure to cytotoxic or pelvic irradiation  for malignancy, taking herbal drugs or other hormonal agents | 20 women with POR | 36.6±4.2 | ELISA | 0.4±0.2 | 0.84±0.2* |
| Vlahos N ^[40]^ | 2015 | FSH＜9.5 mIU/ml，AMH＞2 ng/ml，at least one previous cycle of ovarian  stimulation with more than 3 oocytes retrieved | 48 patients diagnosed with poor response | 39.67±0.54 | Beckman Coulter ELISA | 1.47±0.68 | 1.63±0.76* |
| Yilmaz N ^[41]^ | 2013 | Patients with other infertility factors | 41 patients with DOR | 33.78±4.11 | Gen II ELISA | 0.43±0.31 | 0.79±0.52* |
| Gleicher N ^[42]^ | 2010 | Age-specific FSH less than cut-offs; universal AMH concentrations above the corresponding age limit | 120 patients with DOR | 39±3.1 | MIS/AMH ELISA | 0.22±0.22 | 0.35±0.03* |
| Kara M ^[43]^ | 2014 | AMH ＞1 ng/ml or FSH ＜15 IU/l and antral follicle count ＞4 on day 2  of the menstrual cycle | 104 women undergoing IVFI/CSI due to infertility and DOR | 30.97±5.76 | ELISA | 0.50±0.2 | 0.51±0.26 |
| Elprince M ^[44]^ | 2020 | (1) exposed to cytotoxic or radiotherapy for pelvic malignancies,  (2) on herbal or hormonaltherapy, (3)  with cystectomy or oophorectomy, (4) cases contraindicated  to be supplemented with DHEA (5) Hyperprolactinemia,  (6) polycystic ovaries syndrome, (7) severe male factor infertility, and (8) history of previous IVF cycles. | 25 infertile  women with POR | 36.52±2.26 | ELISA | 0.81±0.14 | 0.996±0.17 |
| Hu Q ^[45]^ | 2017 | PCOS, leiomyoma, endometriosis, metabolic disorders, or a history of ovarian or pelvic surgery, pelvic irradiation,  hormonal treatment or DHEA supplementation | 53 DOR women | 33.28±3.13 | AnshLabs ELISA | 0.87±0.20 | 0.96±0.16* |

AMH: anti-Mullerian hormone; FSH: follicle stimulating hormone; DOR/POR: diminished ovarian reserve / poor ovarian response; DHEA: dehydroepiandrosterone; ELISA, enzyme-linked immunosorbent assay; DHEA (Regular) :75Mg DHEA take orally 3 times a day. *: Before vs. After P < 0.05; Serum AMH level: Mean ± SD or media (95%CI); Age: Mean ± SD or media (95%CI).
