# Supplementary material for: The effect of medication on serum anti-müllerian hormone (AMH) levels in women of reproductive age: a meta-analysis
Source: BMC Endocr Disord. 2022 Jun 14;22:158. doi: 10.1186/s12902-022-01065-9 (PMC9195431; doi:10.1186/s12902-022-01065-9)
Supplement: Supplementary file 5 — Additional file 5: Table S5. The characteristics of the studies included for qualitative analyses. [file 12902_2022_1065_MOESM5_ESM.docx]

**TABLE S5** The characteristics of the studies included for qualitative analyses.

| **Study** | **Year** | **inclusion criteria** | **Population** | **Age**  **(range, mean or media)** | **AMH assay** | **Serum AMH level**  **(ng/ml)** | |
| --- | --- | --- | --- | --- | --- | --- | --- |
|  |  |  |  |  |  | **Before** | **After** |
| Lerchbaum E ^[46]^ | 2021 | Clinical and/or biochemical hyperandrogenism, polycystic ovaries, and/or oligo-/anovulation. | PCOS women (n=80) | 35.7±8.9 | MIS/AMH  ELISA | 9±8.1 | 9±8.5 |
| Naderi Z ^[47]^ | 2018 | 25(OH) D insufficiency  or deficiency (＜30 ng/ml); AMH ＜0.7ng/ml; ＞ 35 years; regular menstrual cycle; normal genitalia and pelvic ultra sound, enteral follicles of ＜4 in the cycle day of  3–5; normal  hysterosalpingography (HSG) and normal semen analysis | 30 infertile women | 37.6±2.65 | ECL | 0.51±0.28 | 1.05±0.64* |
| Dennis N.A ^[48]^ | 2017 | Regular menstrual cycles, not pregnant, had not breastfed within the previous 2 months, not taken Vit D supplements, not travelled to the Northern hemisphere, nor used sun-beds | 27 young healthy women | 21.7±1.1 | Gen II ELISA | 5.59±4.2 | 4.48±3.92* |
| Taheri M ^[49]^ | 2015 | vitamin D deficiency (25(OH)D <75 nmol L -1), non-PCOS | 91 reproductive women | 18-35 | ELISA | 1.97±1.61 | 3.49±1.75* |
| Irani M ^[50]^ | 2013 | 25(OH) D <20 ng/mL | 15 reproductive women | 16-46 | NA | 1.8±0.4 | 2.7±0.6* |
| Wong H.Y. Q ^[51]^ | 2018 | not using hormonal medications or pregnant in the past 3  months | PCOS women (n=15) | 30 (27-34) | liquid chromatography–tandem mass spectrometry | 6.6±3.6 | 10.7±7 |
| Wong H.Y. Q ^[51]^ | 2018 | not using hormonal medications or pregnant in the past 3  months | Non-PCOS women (n=15) | 29 (25-31) | liquid chromatography–tandem mass spectrometry | 3.4±2.2 | 4.4±2.3* |
| Cappy H ^[52]^ | 2016 | contraindication to vitamin D supplementation; having anongoing supplementation or having received a vitamin D supple-mentation in the previous 3 months | NOR women (n=27) | 30.8±5.4 | MIS/AMH ELISA | 3.01±1.05 | 3.33±1.58* |
| Cappy H ^[52]^ | 2016 | Same as above | PCOS women (n=23) | 27.1±4.4 | MIS/AMH ELISA | 10.04±9.45 | 9.59±8.76 |

PCOS: Polycystic Ovary Syndrome; NOR: normal ovarian reserve; VD: vitamin D; ELISA, enzyme-linked immunosorbent assay; ECL: Electro Chemi-Luminescence; VD (Regular): vitamin D capsules containing 20,000-50,000 units administered weekly; *: Before vs. After P < 0.05; Serum AMH level: Mean ± SD or media (95%CI); Age: Mean ± SD or media (95%CI); NA: not available.
