# Supplementary material for: The effect of medication on serum anti-müllerian hormone (AMH) levels in women of reproductive age: a meta-analysis
Source: BMC Endocr Disord. 2022 Jun 14;22:158. doi: 10.1186/s12902-022-01065-9 (PMC9195431; doi:10.1186/s12902-022-01065-9)
Supplement: Supplementary file 6 — Additional file 6: Table S6. The characteristics of the studies included for qualitative analyses. [file 12902_2022_1065_MOESM6_ESM.docx]

**TABLE S6** The characteristics of the studies included for qualitative analyses.

| **Study** | **Year** | **Exclusion criteria** | **Population** | **Age**  **(range, mean or media)** | **AMH**  **Assay** | **BMI** | **Serum AMH level**  **(ng/ml)** | |  |
| --- | --- | --- | --- | --- | --- | --- | --- | --- | --- |
|  |  |  |  |  |  |  | **Before** | **After** |  |
| Aulona Gaba ^[53]^ | 2019 | undergone anyother kind of ovarian stimulation or laparoscopic ovarian drilling | 89 anovulatory, infertile women with PCOS | 29.1 (26.4-33.3) | ELISA | 25.8 (23.3-29.7) | 8.5±5.8 | 5.2±2.7* | |
| MarleneHager ^[54]^ | 2019 | (i) use of co-medications, i.e., metformin, myo-inositol, cortisone/  prednisolone, or any study-specific medications; (ii) any ovarian stimulation within the last 3  months; (iii) women who had become pregnant after the second CC cycle or had chosen not to  undergo any treatment in the subsequent cycle | Anovulatory PCOS patients  (n=41) | 30.2±5.9 | ELISA | 25.6±5.0 | 8.08±4.27 | 7.17±3.37* | |
| Stylianos Vagios ^[55]^ | 2021 | Cycles from women without documented pre-treatment serum AMH levels | 295 PCOS women | 32.5 (30.8–34.8) | MIS/AMH ELISA | 24.6 (22.3-29.4) | 10.1±6.6 | 9.2±7.2* | |
| Vagios S ^[56]^ | 2019 | Non-PCOS women | 172 women with PCOS | Not stated | Not stated | Not stated | 10.5±6.8 | 9.3±7.4* | |
| Gülşen MS ^[57]^ | 2019 | with a history of Cushing’s syndrome, type 2 diabetes  mellitus, hyperprolactinemia, congenital adrenal hyperplasia, thyroid  dysfunction, and ovarian surgery | 63 patients diagnosed as PCOS | 26.6±4.1 | Immunotech ELISA | 25.1±5.25 | 8.06±3.8 | 7.6±4.4* | |
| Xi W ^[58]^ | 2016 | Patients＞35 years；BMI＞30 kg/m with previous ovulation inductionor partners with normal semen parameters. have hyperprolactinemia,Cushing’s syndrome, congenital adrenal hyperplasia or androgen-secreting tumors. | 81 anovulatory women with PCOS | 26.98±2.48 | Immunotech ELISA | 23.53±2.81 | 6.22±2.8 | 4.81±2.06* | |
| Mahran A ^[59]^ | 2012 | Non-PCOS women | 60 anovulatory women with PCOS | Not stated | Not stated | Not stated | 3.9±0.4 | 3.2±0.3* | |
| El-Halawaty S ^[60]^ | 2007 | bilateral tubal block, organic uterine or ovarian pathology ，partners with normal semen parameters， hypo/hyperthyroidism, hyperprolactinaemia  and Cushing’s  syndrome | subfertile women with PCOS (n=68) | 28.21±4.8 | DSL ELISA | 36.7±5.75 | 3.31±3.14 | 4.38±3.30* | |

PCOS: Polycystic Ovary Syndrome; ELISA, enzyme-linked immunosorbent assay; DSL: Diagnostic Systems Laboratories; The unit of BMI: Kg/m^2^; CC: Clomiphene; CC(Regular): 50mg CC administered per day; M: months; *: Before vs. After P < 0.05; Serum AMH level: Mean ± SD or media (95%CI); BMI: Mean ± SD or media (95%CI).
