# Supplementary material for: The effect of medication on serum anti-müllerian hormone (AMH) levels in women of reproductive age: a meta-analysis
Source: BMC Endocr Disord. 2022 Jun 14;22:158. doi: 10.1186/s12902-022-01065-9 (PMC9195431; doi:10.1186/s12902-022-01065-9)
Supplement: Supplementary file 7 — Additional file 7: Table S7. The characteristics of the studies included for qualitative analyses. [file 12902_2022_1065_MOESM7_ESM.docx]

**TABLE S7** The characteristics of the studies included for qualitative analyses.

| **Study** | **Year** | **Exclusion criteria** | **Population** | **Age**  **(range, mean or media)** | **AMH assay** | **Study type** | **Serum AMH level**  **(ng/ml)** | |  |
| --- | --- | --- | --- | --- | --- | --- | --- | --- | --- |
|  |  |  |  |  |  |  | **Before** | **After** |  |
| Vagios S ^[55]^ | 2021 | women without documented pre-treatment serum AMH levels | 180 patients with the diagnosis of PCOS | 32.1 (30.5–34.2) | MIS/AMH ELISA | self- control | 11.0±7.5 | 10.7±5.4* | |
| Vagios S ^[56]^ | 2019 | Non-PCOS women | 172 women with PCOS | Not stated | Not stated | self- control | 10.5±6.8 | 11.4±5.5* | |
| Andersen C.Y ^[61]^ | 2008 | undergoing IVF/ICSI treatment without received androgen priming before controlled ovarian  hyperstimulation. | 45 infertile women | Not stated | DSL ELISA | self- control | 1.6±0.2 | 1.5±0.4* | |

PCOS: Polycystic Ovary Syndrome; ELISA, enzyme-linked immunosorbent assay; DSL: Diagnostic Systems Laboratories; LET: letrozole; LET(Regular): 2.5mg LET administered per day on 3-5 days of menstruation, for 3 months; *: Before vs. After P < 0.05; Serum AMH level: Mean ± SD or media (95%CI).
