# Supplementary material for: The effect of medication on serum anti-müllerian hormone (AMH) levels in women of reproductive age: a meta-analysis
Source: BMC Endocr Disord. 2022 Jun 14;22:158. doi: 10.1186/s12902-022-01065-9 (PMC9195431; doi:10.1186/s12902-022-01065-9)
Supplement: Supplementary file 8 — Additional file 8: Table S8. Newcastle-Ottawa Scale for assessing quality of interventional cohort studies. [file 12902_2022_1065_MOESM8_ESM.docx]

**Table S8**. Newcastle-Ottawa Scale for assessing quality of interventional cohort studies.

| author | year | Represent-  ativeness of exposed cohort | Selection of non-exposed cohort | Exposure assessment | Outcome of interest not present at start of study | Compara-  bility  of cohorts | Outcome assessment | Adequacy of length of time before follow-up | Adequacy of follow-up of cohorts | Total  quality  scores |
| --- | --- | --- | --- | --- | --- | --- | --- | --- | --- | --- |
| Kucera R | 2016 | ★ | ★ | ★ | ★ | ★ | ★ | ★ |  | 7 |
| Johnson LN | 2014 | ★ | ★ | ★ | ★ | ★ | ★ | ★ |  | 7 |
| Kallio S | 2013 |  | ★ | ★ | ★ | ★★ | ★ | ★ |  | 7 |
| Bentzen JG | 2012 | ★ | ★ | ★ | ★ | ★ | ★ | ★ | ★ | 8 |
| Deb S | 2012 | ★ | ★ | ★ | ★ | ★ | ★ | ★ |  | 7 |
| Li HW | 2011 | ★ | ★ |  | ★ | ★ | ★ | ★ |  | 6 |
| Arbo E | 2007 |  | ★ | ★ | ★ | ★ | ★ | ★ |  | 6 |
| Somunkiran A | 2007 |  | ★ | ★ | ★ | ★ | ★ | ★ | ★ | 7 |
| Saleh BO | 2015 | ★ | ★ | ★ | ★ | ★ | ★ | ★ | ★ | 8 |
| Madsen HN | 2015 | ★ | ★ | ★ | ★ | ★ | ★ | ★ |  | 7 |
| Neagu M | 2012 |  | ★ | ★ | ★ | ★ |  | ★ | ★ | 6 |
| Nascimento AD | 2013 | ★ | ★ | ★ | ★ | ★ | ★ | ★ | ★ | 8 |
| Tomova A | 2011 | ★ | ★ | ★ | ★ | ★ | ★ | ★ | ★ | 8 |
| Panidis D | 2011 | ★ | ★ | ★ | ★ | ★ | ★ | ★ |  | 7 |
| Carlsen SM | 2009 | ★ | ★ | ★ | ★ | ★ | ★ | ★ |  | 7 |
| Piltonen T | 2005 | ★ | ★ | ★ | ★ | ★ | ★ | ★ |  | 7 |
| Chhabra N | 2018 | ★ | ★ | ★ | ★ | ★ |  | ★ |  | 6 |
| Dawoud, Z | 2018 |  | ★ | ★ | ★ | ★ | ★ | ★ |  | 6 |
| Wiweko, B | 2017 | ★ | ★ | ★ | ★ | ★ | ★ | ★ | ★ | 8 |
| Foroozanfard, F | 2015 |  | ★ | ★ | ★ | ★ | ★ | ★ | ★ | 7 |
| Su HI | 2013 | ★ | ★ | ★ | ★ | ★ | ★ | ★ |  | 7 |
| Drakopoulos P | 2019 | ★ | ★ | ★ | ★ | ★ | ★ | ★ |  | 7 |
| Cai J | 2018 |  | ★ | ★ | ★ | ★ | ★ | ★ |  | 6 |
| Mocciaro, R | 2016 | ★ | ★ | ★ | ★ | ★ |  | ★ | ★ | 7 |
| Marschalek, J | 2015 |  | ★ | ★ | ★ | ★ | ★ | ★ |  | 6 |
| Agarwal, R | 2017 | ★ | ★ | ★ | ★ | ★ | ★ | ★ | ★ | 8 |
| Tsui, K.-H | 2015 | ★ | ★ | ★ | ★ | ★ | ★ | ★ |  | 7 |
| Vlahos, N | 2015 | ★ | ★ | ★ | ★ | ★ | ★ | ★ |  | 7 |
| Yilmaz, N | 2013 |  | ★ | ★ | ★ | ★ | ★ | ★ |  | 6 |
| Gleicher, N | 2010 | ★ | ★ | ★ | ★ | ★ | ★ | ★ | ★ | 8 |
| Kara M | 2014 | ★ | ★ | ★ | ★ | ★ |  | ★ |  | 6 |
| Elprince M | 2020 | ★ | ★ | ★ | ★ | ★ | ★ | ★ | ★ | 8 |
| Hu Q | 2017 | ★ | ★ | ★ | ★ | ★ | ★ | ★ |  | 7 |
| Lerchbaum, E | 2021 |  | ★ | ★ | ★ | ★ | ★ | ★ | ★ | 7 |
| Naderi, Z | 2018 | ★ | ★ | ★ | ★ | ★ |  | ★ |  | 6 |
| Dennis,N.A | 2017 | ★ | ★ | ★ | ★ | ★ | ★ | ★ | ★ | 8 |
| Taheri, M | 2015 | ★ | ★ | ★ | ★ | ★ | ★ | ★ |  | 7 |
| Irani, M | 2013 |  | ★ | ★ | ★ | ★ |  | ★ | ★ | 6 |
| Wong, H.Y. Q | 2018 | ★ | ★ | ★ | ★ | ★ | ★ | ★ |  | 7 |
| Cappy H | 2016 |  | ★ | ★ | ★ | ★ | ★ | ★ |  | 6 |
| Aulona Gaba | 2019 | ★ | ★ | ★ | ★ | ★ | ★ | ★ |  | 7 |
| MarleneHager | 2019 | ★ | ★ | ★ | ★ | ★ | ★ | ★ | ★ | 8 |
| Stylianos Vagios | 2021 |  | ★ | ★ | ★ | ★ | ★ | ★ | ★ | 7 |
| Vagios, S | 2019 |  | ★ | ★ | ★ | ★ |  | ★ | ★ | 6 |
| Gülşen, MS | 2019 | ★ | ★ | ★ | ★ | ★ | ★ | ★ | ★ | 8 |
| Xi, W | 2016 | ★ | ★ | ★ | ★ | ★ | ★ | ★ |  | 7 |
| Mahran, A | 2012 | ★ | ★ | ★ | ★ | ★ |  | ★ |  | 6 |
| El-Halawaty S | 2001 |  | ★ | ★ | ★ | ★ | ★ | ★ |  | 6 |
| Vagios S | 2021 | ★ | ★ | ★ | ★ | ★ | ★ | ★ |  | 7 |
| Vagios S | 2019 |  | ★ | ★ | ★ | ★ |  | ★ | ★ | 6 |
| Andersen,C.Y | 2008 | ★ | ★ | ★ | ★ | ★ | ★ | ★ |  | 7 |

a. A study can be awarded a maximum of one star for each numbered item except for the item, “Comparability of cohorts”. b. A maximum of two stars can be awarded for “Comparability of cohorts”.
